# N6-Methyladenosine RNA Methylation in Cardiovascular Diseases

**DOI:** 10.3389/fcvm.2022.887838

**Published:** 2022-04-29

**Authors:** Chi Liu, Lei Gu, Wenjuan Deng, Qianchao Meng, Nan Li, Guifeng Dai, Suli Yu, Hong Fang

**Affiliations:** ^1^Department of Cardiology, Tongji Hospital, School of Medicine, Tongji University, Shanghai, China; ^2^Department of Geriatrics Center, National Clinical Research Center for Aging and Medicine, Jing’an District Central Hospital of Shanghai, Fudan University, Shanghai, China; ^3^Department of Internal Medicine, Shanghai Shende Hospital, Shanghai, China; ^4^Innovation Research Institute of Traditional Chinese Medicine, Shanghai University of Traditional Chinese Medicine, Shanghai, China; ^5^Department of Hand and Upper Extremity Surgery and Limb Function Reconstruction Center, Jing’an District Central Hospital, Shanghai, China

**Keywords:** N6-methyladenosine, cardiovascular diseases, biomarkers, therapeutic target, molecular mechanism

## Abstract

N6-methyladenosine (m6A) modification is the most universal and abundant post-transcriptional modification of eukaryotic RNA and occurs mainly at the consensus motif RR (m6A) CH (R = A or G, H = A, C, or U) in long internal exons, near stop codons, or in the 3′ untranslated region (UTR). “Writers,” “erasers,” and “readers” are responsible for the occurrence, removal, and recognition of m6A modification, respectively. Substantial evidence has shown that m6A RNA modification can exert important functions in physiological and pathological processes. Cardiovascular diseases (CVDs) are a wide array of disorders affecting heart or vessels, including atherosclerosis (AS), hypertension (HT), ischemia/reperfusion (I/R) injury, myocardial infarction (MI), stroke, cardiac hypertrophy, heart failure (HF), and so on. Despite the advances in lipid-lowering drugs, antihypertensives, antiplatelet agents, and anticoagulation therapy, CVDs are still the leading cause of death worldwide. Recent studies have suggested that m6A modification of RNA may contribute to the pathogenesis of CVDs, providing a novel research insight for CVDs. Herein, we provide an up-of-date summarization of the molecular mechanism of m6A and the roles of m6A in different types of CVDs. At last, we propose that m6A might be a potiential biomarker or therapeutic target for CVDs.

## Introduction

Decades of research has revealed that epigenetic mechanisms, including DNA methylation, histone methylation and acetylation, chromatin remodeling, and non-coding RNA, play critical roles in physiological and pathological processes besides heredity and environment. Epigenetic mechanisms are reversible, heritable, and are affected by the external environment. In addition to those modifications occurring in DNA and histone, RNA modification was also reported to regulate biological processes in recent years. Among more than 170 types of RNA modification, the N6-methyladenosine (m6A) modification, which was first reported in Novikoff hepatoma cells in the 1970s, is the most universal and abundant internal modification of eukaryotic messenger RNA (mRNA) (1). m6A modification exists in more than 7,000 mRNAs and in 0.1–0.4% of adenosines in mammalian cells (2, 3). Studies have suggested that m6A can not only occur in mRNA, but in small nucleolar RNA (snRNA), microRNA (miRNA), long non-coding RNA (lncRNA), circular RNA (circRNA), ribosomal RNA (rRNA), and transfer RNA (tRNA) (3–7).

Cardiovascular diseases (CVDs) are a wide array of disorders affecting heart or vessels, including atherosclerosis (AS), hypertension (HT), ischemia/reperfusion (I/R) injury, myocardial infarction (MI), stroke, cardiac hypertrophy, heart failure (HF), and so on. There are some causal links among various CVDs. Plaque of AS can cause vascular stenosis which contributes to ischemia and even MI (8). I/R injury destroys cardiomyocytes by inducing oxidative stress and releasing oxidative free radicals (9), which further leads to MI and HF (10). Moderate cardiac hypertrophy is considered as a physiological response adapting to pro-hypertrophic stimuli; however, sustained hypertrophy leads to cardiac decompensation and consequently HF (10). HT is a great risk factor for other CVDs such as stroke, MI, and HF (11). In addition, many other diseases such as the current pandemic of coronavirus disease 2019 (COVID-19) also lead to severe cardiovascular symptoms like disseminated intravascular coagulation (DIC) and low blood pressure. Despite the advances in lipid-lowering drugs, antihypertensives, antiplatelet agents, and anticoagulation therapy, CVDs are still the leading cause of death worldwide. Recent studies have suggested that m6A RNA modification may contribute to the pathogenesis of CVDs, providing a novel research insight for CVDs. Herein, we provide an up-of-date summarization of the molecular mechanism of m6A and the roles of m6A in different types of CVDs. At last, we propose that m6A might be a potential biomarker or therapeutic target for CVDs.

## m6A RNA Methylation

**FIGURE 1:**
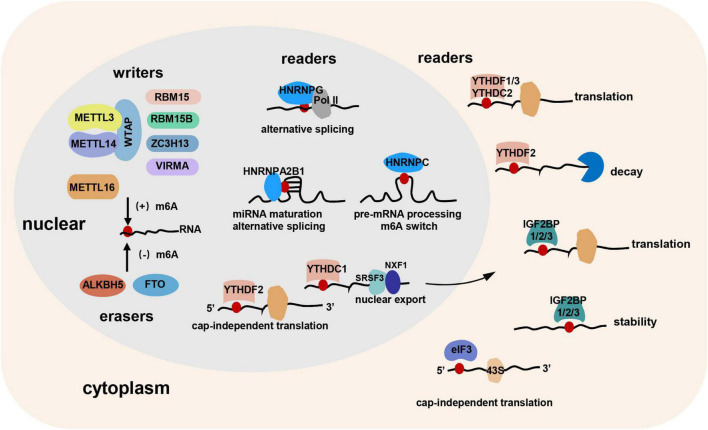
The “writers,” “erasers,” and “readers” of m6A RNA modification. “Writers,” “erasers,” and “readers” are responsible for the occurrence, removal, and recognition of m6A modification, respectively. METTL3, METTL14, and WTAP constitute the core component of the methyltransferase complex. RBM15, RBM15B, ZC3H13, and VIRMA may bind to the complex. In addition, METTL16 might be an independent m6A methyltransferase. FTO and ALKBH5 are the two m6A demethylases. m6A “readers” contain YTH domain family, IGFBP family, hnRNP family, eIF3, and so on. Cytoplasmic “readers,” including YTHDF1, YTHDF2, YTHDF3, YTHDC2, IGF2BP1/2/3, and eIF3 can regulate the translation, decay, stability, and cap-independent translation of RNA. Nuclear “readers,” including YTHDC1, YTHDF2, HNRNPC, HNRNPG, and HNRNPA2B1 can modulate miRNA maturation, alternative splicing, cap-independent translation, nuclear export, and pre-mRNA processing.

Most of the studies considered the m6A as a post-transcriptional modification of RNA (12, 13). However, Slobodin et al. (14) and Xu et al. (15) demonstrated that m6A RNA modification occurs co-transcriptionally. Rapid development of bioinformatic analyses and high-throughput sequencing technologies have identified that m6A modification occurs mainly at the consensus motif RR (m6A) CH (R = A or G, H = A, C, or U) in long internal exons, near stop codons, or in the 3′ untranslated region (UTR) (3, 16). “Writers,” “erasers,” and “readers” are responsible for the occurrence, removal, and recognition of m6A RNA modification, respectively. m6A modification has been shown to alter RNA secondary structures or be recognized by “readers” to regulate the metabolism of methylated mRNAs, thereby exerting various functions (Figure 1).

### Writers

m6A “writers” are acted by the methyltransferase complex. Methyltransferase-like 3 (METTL3), METTL14, and Wilms tumor 1-associated protein (WTAP) constitute the core component of the complex. METTL3 was the first discovered methyltransferase subunit mediating m6A methylation through S-adenosylmethionine (SAM)-binding motif (17). Afterward, METTL14, also containing SAM-binding motif, was reported forming a heterodimeric complex with METTL3 and synergistically catalyzing m6A RNA methylation with METTL3 in mammals (18). However, Wang et al. (19) reported an inconsistent conclusion. They found that the catalytic subunit of METTL3 is critical while that of METTL14 is dispensable, and METTL14 mainly plays a structural role for substrate recognition. WTAP, although showing no methyltransferase activity, can interact with METTL3/METTL14 complex and be responsible for the localization into nuclear speckles and recruitment to mRNA targets of METTL3/METTL14 complex, thereby affecting m6A methylation (18). In addition, RNA-binding motif protein 15 (RBM15), RBM15B, zinc finger CCCH-type containing 13 (ZC3H13), and vir-like m6A methyltransferase associated (VIRMA, also known as KIAA1429) were also reported binding to the m6A-methylation complex to regulate RNA methylation (20–22), but the detailed mechanisms of action need to be further explored. In addition, a recent study proposed that METTL16 is also an independent m6A RNA methyltransferase preferring a UAC (m6A) GAGAA sequence in the bulge of a stem-loop structured RNA (23), and plays a critical role in splicing regulation (24).

### Erasers

The discovery of m6A demethylases reveals that m6A methylation is a dynamic and reversible process, and thus m6A demethylases are regarded as the “erasers” of m6A methylation. The fat mass and obesity-associated protein (FTO) and AlkB homolog 5 (ALKBH5) are the two m6A demethylases reported so far. They both belong to the AlkB family proteins and non-heme Fe (II)/alpha-ketoglutarate-dependent dioxygenases. Existing studies demonstrated that the deficient of FTO or ALKBH5 led to an elevated level of m6A in mRNAs (25, 26). Mechanistically, they first oxidizes m6A to N6-hydroxymethyladenosine (hm6A), and hm6A is further oxidized to N6-formyladenosine (f6A) which is at last hydrolyzed to adenine (27, 28). The expression patterns of FTO and ALKBH5 are distinct among tissues, and interaction with different protein partners leads to different substrate repertoire of FTO and ALKBH5 (29). Through demethylation of m6A-modified RNA, FTO, and ALKBH5 can regulate RNA transfer and metabolism.

### Readers

YTH (YT521-B homology) domain family proteins containing YTHDF1-3 and YTHDC1-2 constitute a class of m6A “reader,” and can regulate RNA translation, degradation, and transport by forming an aromatic cage to specifically recognize m6A. Liu et al. (30) found that YTHDF1 promoted the translation of m6A-modified EIF3C (a subunit of the protein translation initiation factor EIF3) and thus increased the overall translational level, thereby facilitating ovarian cancer progression. Similarly, Zhuang et al. (31) demonstrated that YTHDF1 promoted the translation of m6A-modified Roundabout (Robo) family axon guidance receptor Robo3.1, and thus regulated pre-crossing axon guidance. Zhou et al. (32) found that heat shock stress induced nuclear localization of YTHDF2, which protected 5′UTR m6A methylation of stress-induced transcripts from demethylation by FTO and further promoted translation initiation independent of 5′ cap. However, another study demonstrated that YTHDF2 facilitated the degradation of m6A-modified RNA by recruiting the carbon catabolite repressor 4–negative on TATA (Ccr4-Not) deadenylase complex (33). Li et al. (34) suggested that YTHDF3 may facilitate the translation of targeted m6A-modified mRNAs by cooperating with YTHDF1 and recruiting other translational effectors. YTHDC1 was reported mediating the nuclear export of methylated mRNA by facilitating these methylated mRNA binding to serine/arginine-rich splicing factor 3 (SRSF3) and nuclear RNA export factor 1 (Nxf1) (35). RNA helicase-containing YTHDC2 was reported promoting translational efficiency of the m6A-modified coding region by unfolding mRNA secondary structures (36).

IGFBP proteins including IGF2BP1/2/3 contain two RNA recognition motif (RRM) domains and four K homology (KH) domains, and can regulate RNA localization, translation, and stability (37). Interestingly, Huang et al. (38) demonstrated that IGF2BPs preferentially bound to the UGGAC consensus sequence which contains the m6A core motif GGAC. They also showed that IGF2BPs can recognize m6A to increase mRNA stability and translation through KH domain at a global level, thereby exerting oncogenic functions.

The third class of m6A “reader” proteins is heterogeneous nuclear ribonucleoprotein (hnRNP) family including hnRNPG, hnRNPC, and hnRNPA2B1. Liu et al. (39) found that rather than recognizing m6A directly, hnRNPC selectively bound to m6A-modified transcripts to promote pre-mRNA processing through “m6A-switch” mechanism, in which m6A modification unfolded the RNA hairpin structure to facilitate the binding of hnRNPC. Zhou et al. (40) reported that hnRNPG can bind m6A near splice sites of nascent pre-mRNA co-transcriptionally to modulate RNA polymerase II (RNAPII) occupancy and alternative splicing. HNRNPA2B1 was proposed to be a nuclear m6A “reader” to mediate primary microRNA maturation and alternative splicing (41).

Eukaryotic initiation factor 3 (eIF3) may also serve as an m6A “reader” to initiate the cap-independent translation. Meyer et al. (42) showed that m6A sites in the 5′ UTR of mRNA can directly bind to eIF3, which can further recruit the 43S complex to initiate the cap-independent translation.

Collectively, m6A modification is a process responding rapidly to external stimuli. Upon external stimuli, m6A marks transcripts encoding critical regulators to facilitate their transfer and metabolism, and thereby enables cells to switch transcriptomes to adapt to the cell fate transition (43). Shi et al. (29) suggested that the function of m6A might be modulated by multiple layers of contexts. First, distinct cellular signaling or external stimuli may determine the various functions of m6A. Second, expression levels and subcellular localization of m6A effectors also matter. For example, changes in post-translational modifications of m6A effectors such as SUMOylation, phosphorylation, and ubiquitination or interaction with protein partners might regulate the spatial distribution of “writers,” “erasers,” and “readers,” thereby modulating the function of m6A. Third, different locations of m6A sites on an mRNA may have different regulatory functions. Densely populated m6A regions may be more frequently bound by m6A “readers.” Finally, different cell types or cell differentiation and developmental status may also contribute to the varied roles of m6A in physiological status or in diseases. Of note, the mechanisms determining the specificity of m6A effectors have not been fully elucidated, and several recent studies suggested that exploring underlying intrinsic and extrinsic factors will help to understand the nature of m6A as a regulatory system (43, 44).

### Methods to Detect m6A

Current methods for m6A detection are based on RNA chemistry or immunoprecipitation to assess an overall transcriptome-wide m6A level or precise location of m6A sites by virtue of high-throughput sequencing. Dot blot technology (45), colorimetric method (46), and chemical proteomics approach (47) are common approaches to observe overall m6A changes in transcriptome-wide level. They do achieve the quantitation or semi-quantitation, but fail to precisely locate the m6A sites. At present, methylated RNA immunoprecipitation (MeRIP)-seq (16) and m6A-seq (3) are the most used approaches for the detection of m6A sites. Total RNA is first extracted and fragmented, and then these fragmented RNAs are immunoprecipitated by m6A antibody followed by next-generation sequencing. However, the approaches can only detect m6A peaks but not acquire the precise position of m6A residues. To overcome the shortcoming, several approaches such as m6A individual-nucleotide-resolution cross-linking and immunoprecipitation (miCLIP) (48), m6A level and isoform-characterization sequencing (m6A-LAIC-seq) (49), site-specific cleavage and radioactive labeling followed by ligation-assisted extraction and thin-layer chromatography (SCARLET) (50), and MAZTER-seq (51) were newly developed to achieve more precise location even at single-nucleotide resolution.

## m6A in Vascular Smooth Muscle Cells

The “writers” of m6A were reported participating in the proliferation and migration of vascular smooth muscle cells (VSMCs). For example, Zhu et al. (52) illustrated that WTAP inhibited the proliferation and migration of VSMCs through m6A-mediated expression upregulation of p16, a cyclin-dependent kinase inhibitor. In addition, a study showed that METTL14 and WTAP might promote VSMCs apoptosis by increasing the expression of apoptosis-related genes in an m6A-dependent manner (53). Moreover, Chen et al. (54) showed that METTL14 facilitated the calcification of indoxyl sulfate-treated human artery smooth muscle cells (HASMCs) by methylating the artery preventing transcript *Klotho* and promoting its degradation.

In contrast to methyltransferases, demethylases were reported to promote the proliferation and migration of VSMCs. For example, Ma et al. (55) demonstrated that both FTO overexpression and angiotensin II (AngII)-induced FTO expression promoted the proliferation and migration of VSMCs. Mechanistically, FTO increased Krüppel-like Factor 5 (KLF5) expression by demethylating m6A modification of *KLF5* as well as enhancing glycogen synthase kinase-3 beta (GSK3β) signaling activation in VSMCs, and thus regulated VSMC phenotypic switching, highlighting the potential of FTO as a therapeutic target for aortic dissecting aneurysm (ADA)-associated diseases. Consistently, Huo et al. (56) also showed that FTO promoted AngII-induced VSMC proliferation and inflammatory response by demethylating m6A modification of nuclear receptor subfamily 4, group A, member3 (NR4A3) mRNA.

Different m6A “readers” were reported exerting distinct functions in the proliferation and migration of VSMCs. Yuan et al. (57) found that YTHDC2 positively regulated *circYTHDC2* stability in an m6A-dependent manner, which further reduced the expression of ten-eleven translocation 2 (TET2) a modulator of phenotypic plasticity of VSMCs (58), thereby promoting the proliferation and migration of VSMCs. In addition, Zhang et al. (59) showed that IGF2BP2 increased smooth muscle 22 alpha (SM22α) mRNA stability by serving as the “reader” of m6A-modified *SM22*α, which further inhibited the proliferation and migration of VSMCs and alleviated intimal hyperplasia in T2DM, revealing the protective role of IGF2BP2 in VSMCs dysfunction.

## The Roles of m6A in Cardiovascular Diseases

m6A RNA modification has been reported contributing substantially to the pathogenesis of CVDs. Herein, we provide an up-of-date summarization of the roles of m6A regulators in different types of CVDs and corresponding mechanisms (Table 1).

**TABLE 1:** The roles of m6A in cardiovascular diseases.

CVDs	m6A regulator	Target genes	Function	References
AS	METTL14	Pri-miR-19a	Promotes the maturation of pri-miR-19a into miR-19a, thereby enhancing the proliferative and invasive ability of ASVECs	(60)
	METTL14	*FOXO1*	Promotes the expression of *VCAM-1* and *ICAM-1* by increasing FOXO1, thus inducing inflammation in TNF-α-induced HUVECs	(61)
	METTL3	*NLRP1 KLF4*	Exerts proinflammatory effects in HUVECs or MAECs by increasing *NLRP1*, reducing *KLF4*, and activating NF-κB	(62)
	METTL3	*LRP6 DVL1*	Promotes the translation of LRP6 and DVL1 in HUVECs under hypoxic stress, thus exerting an angiogenic role	(63)
	METTL3	*EGFR*	Promotes *EGFR* degradation, thus alleviating endothelial atherogenic progression	(64)
HT	m6A-SNPs	*rs9847953* *rs197922*,…	BP-associated m6A-SNPs are associated with diastolic and systolic BP	(66)
	*MC4R* m6A-SNP	*rs17782313*	*MC4R* m6A-SNP *rs17782313* is negatively correlated with mean BP and diastolic BP in men with HT	(67)
I/R injury	ALKBH5	*WNT5A*	Negatively regulates the post-ischemic angiogenesis by promoting *WNT5A* degradation in CMECs	(68)
	FTO	*SERCA2a*	Promotes SERCA2a expression, thereby maintaining calcium homeostasis and improving energy metabolism in H/R cardiomyocytes	(69)
	FTO	*Mhrt*	Inhibits the apoptosis of H/R-treated cardiomyocytes by reducing m6A modification of *Mhrt* and increasing its expression	(70)
	METTL3	Pri-miR-143-3p	Facilitates cardiomyocyte pyroptosis and I/R injury by promoting pri-miR-143-3p maturation and further reducing PRKCE expression	(71)
	WTAP	*ATF4*	Facilitates ER stress and cell apoptosis by increasing the expression of ATF4, thereby exacerbating myocardial I/R injury	(72)
	METTL3	miR-873-5p miR-25-3p	Promotes the maturation of miR-873-5p and miR-25-3p, and further activates the PI3K/AKT, thereby suppressing I/R injury	(73)
	METTL14	*WNT1*	Alleviates I/R injury through m6A-mediated expression upregulation of Wnt1 protein and activation of Wnt/β-catenin signaling	(74)
	METTL3 ALKBH5	*TFEB*	METTL3 impedes and ALKBH5 promotes autophagic flux and apoptosis of H/R-treated cardiomyocytes by decreasing *TFEB* expression	(76)
MI	ALKBH5	-	ALKBH5 knockout led to lower serum levels of LDH and CK-MB as well as improved systolic and diastolic functions in acute MI mice	(78)
	METTL3	Pri-miR-143	Promotes pri-miR-143 maturation and further reduces Yap and CTNND1 expression, thereby impeding cardiomyocyte proliferation	(79)
	WTAP	-	WTAP SNP is significantly correlated with the progression of MI based on GWAS analysis	(80)
Cardiac hypertrophy	METTL3	*PARP10*	Accelerates *PARP10* degradation and plays a protective role in cardiac hypertrophy through METTL3-PARP10-GSK3β axis	(81)
	METTL3	-	Aggravates the Ang II-induced cardiac hypertrophy	(84)
	METTL3	-	Regulates cardiac homeostasis	(85)
	YTHDF2	*MYH7*	Inhibits cardiac hypertrophy through m6A-dependent *MYH7* degradation	(86)
	IGF2BP2	miR-133a target mRNA	Forms a complex with AGO2 to facilitate miR-133a’s accumulation on target mRNAs, thereby preventing cardiac hypertrophy	(87)
HF	FTO	-	Cardiomyocyte-specific knockout of *Fto* accelerated the progression of HF in TAC mice	(89)
	FTO	-	Attenuates ischemia-induced cardiac dysfunction in HF mice	(90)
	FTO	-	Mitigates cardiac dysfunction by modulating glycolysis and glucose uptake in TAC-induced HF mice	(91)

*AGO2, argonaute 2; Ang II, angiotensin II; AS, atherosclerosis; ASVECs, atherosclerotic vascular endothelial cells; ATF4, activating transcription factor 4; BP, blood pressure; CK-MB, creatine kinase-MB; CMECs, cardiac microvascular endothelial cells; CTNND1, catenin delta-1; DVL1, disheveled 1; EGFR, epidermal growth factor receptor; ER, endoplasmic reticulum; FOXO1, forkhead box O1; GSK3β, glycogen synthase kinase-3 beta; HF, heart failure; H/R, hypoxia/reoxygenation; HT, hypertension; HUVECs, human umbilical vein endothelial cells; ICAM-1, intercellular adhesion molecule-1; I/R, ischemia/reperfusion; KLF4, krüppel-like factor 4; LDH, lactate dehydrogenase; LRP6, low density lipoprotein receptor-related protein 6; MAECs, mouse aortic endothelial cells; MC4R, melanocortin 4 receptor; Mhrt, myosin heavy chain associated RNA transcript; MI, myocardial infarction; MYH7, myosin heavy chain 7; NLRP1, NOD-like receptor protein 1; PARP10, poly (ADP-ribose) polymerase 10; PRKCE, protein kinase C epsilon; SERCA2a, sarcoplasmic/endoplasmic reticulum calcium ATPase 2a; SNPs, single nucleotide polymorphisms; TAC, transverse aortic constriction; TFEB, transcription factor EB; VCAM-1, vascular cell adhesion molecule-1; WNT5A, WNT family member 5A; Yap, Yes-associated protein.*

### m6A in Atherosclerosis

Several studies showed that methyltransferases such as METTL14 and METTL3 mainly exert angiogenic function in AS. Zhang et al. (60) found that m6A level and METTL14 expression were significantly upregulated in atherosclerotic vascular endothelial cells (ASVECs). They further demonstrated the upregulated METTL14 increased the expression of RNA splicing-related protein DGCR and m6A modification of pri-miR-19a, which promoted the formation of mature miR-19a, thereby enhancing the proliferative and invasive ability of ASVECs. These results reveal that METTL14-m6A-miR-19a axis may be a potential therapeutic target for AS. Jian et al. (61) showed that METTL14 promoted the expression of transcription factor forkhead box O1 (FOXO1) by increasing its m6A modification, which further promoted the transcription of adhesive molecules vascular cell adhesion molecule-1 (*VCAM-1*) and intercellular adhesion molecule-1 (*ICAM-1*), thus inducing endothelial inflammation in TNF-α-induced human umbilical vein endothelial cells (HUVECs), revealing the positive role of METTL14 in vascular endothelial cell inflammation and AS development. Chien et al. (62) showed that METTL3 exerted proinflammatory effects in HUVECs or mouse aortic endothelial cells (MAECs) exposed to pro-atherogenic oscillatory stress (OS) or TNF-α stimulation by upregulating NOD-like receptor protein 1 (*NLRP1*) and downregulating krüppel-like factor 4 (*KLF4*) in m6A-dependent manner as well as activating p65/NF-κB pathway, thereby promoting the adhesion of inflammatory cells and the initiation of AS. Yao et al. (63) demonstrated that METTL3 promoted the translation of Wnt signaling-related proteins low density lipoprotein receptor-related protein 6 (LRP6) and disheveled 1 (DVL1) in YTHDF1-dependent manner in HUVECs under hypoxic stress, thus exerting an angiogenic role. However, Li et al. (64) reported a protective role of METTL3 in AS. They revealed that METTL3 promoted the m6A-dependent degradation of vascular endothelial cell dysfunction-related molecule epidermal growth factor receptor (EGFR) mRNA, thus alleviating endothelial atherogenic progression.

### m6A in Hypertension

An m6A high-throughput sequencing analysis showed that the global m6A level was reduced in the pericytes of spontaneous HT rats, implying that m6A might regulate mammalian HT (65). A study by Mo et al. (66) identified 1,236 blood pressure (BP)-associated m6A-single nucleotide polymorphisms (SNPs) such as *rs9847953* and *rs197922*, and among them, 799 and 761 were associated with diastolic and systolic BP, respectively. Another study by Marcadenti et al. (67) showed that a melanocortin 4 receptor (MC4R) m6A-SNP, *rs17782313*, is negatively correlated with mean BP and diastolic BP in men with HT. Collectively, these results reinforce the potential roles of m6A in BP regulation.

### m6A in Ischemia/Reperfusion Injury

Zhao et al. (68) demonstrated that ALKBH5 negatively regulated the post-ischemic angiogenesis by reducing the m6A level of WNT family member 5A (WNT5A) mRNA and promoting its degradation in cardiac microvascular endothelial cells (CMECs). Deng et al. (69) demonstrated that FTO expression was decreased in H/R-treated cardiomyocytes. FTO decreased the m6A level of sarcoplasmic/endoplasmic reticulum calcium ATPase 2a (SERCA2a) mRNA through demethylation, and thus promoted SERCA2a expression, which can maintain calcium homeostasis and improve energy metabolism in H/R cardiomyocytes. Shen et al. (70) demonstrated that overexpression of FTO inhibited the apoptosis of H/R-treated cardiomyocytes by reducing m6A modification of myosin heavy chain associated RNA transcript (*Mhrt*) a cardiac-specific lncRNA derived from the myosin heavy chain 7 (MYH7) gene antisense strand and increasing its expression. These studies reinforce the protective roles of demethylases in I/R injury.

Wang et al. (71) showed that METTL3 level was upregulated in I/R rats and oxygen-glucose deprivation/reoxygenation (OGD/R)-treated cardiomyocytes. METTL3 promoted miR-143-3p expression through DGCR8-mediated pri-miR-143-3p maturation in an m6A-dependent manner, which further reduced protein kinase C epsilon (PRKCE) expression level, thereby exacerbating cardiomyocyte pyroptosis and I/R injury. In addition, a study showed that WTAP facilitated endoplasmic reticulum (ER) stress and cell apoptosis in both HR-treated human cardiomyocytes and I/R-treated rat hearts by increasing mRNA m6A level of activating transcription factor 4 (ATF4) a transcription factor controlling ER-related gene expression and upregulating its expression, thereby exacerbating myocardial I/R injury (72). However, there are also studies demonstrating the protective roles of m6A “writers” in cardiac I/R injury. Zhao et al. (73) showed that METTL3 level was downregulated in I/R mice hearts and hypoxia/reoxygenation (H/R) cardiomyocytes, and overexpression of METTL3 reduced I/R and H/R-induced cardiomyocyte apoptosis. Mechanistically, METTL3 upregulated the levels of miR-873-5p and miR-25-3p by promoting their maturation, and further activated the PI3K/AKT signaling pathway, thereby suppressing I/R-induced myocardial injury. Pang et al. (74) showed that METTL14 protein levels were increased in mice hearts after I/R. Mice with METTL14 overexpression showed the significantly improved cardiac function, manifested by reduced myocardial infarct size, serum levels of lactate dehydrogenase (LDH), and apoptosis of cardiomyocytes after I/R injury through m6A-mediated expression upregulation of Wnt1 protein and activation of Wnt/β-catenin signaling.

Intriguingly, a study reported that m6A modification plays a role in I/R by modulating autophagy. The authors found that m6A level was increased in cardiomyocytes exposed to H/R and mice hearts after I/R. Overexpressing METTL3 or inhibiting ALKBH5 impeded autophagic flux and apoptosis of H/R-treated cardiomyocytes by enhancing the methylation of transcription factor EB (TFEB), a positive regulator of autophagy (75), and decreasing its expression level. However, no improved manifestations were observed in cardiomyocyte-specific *Mettl3* knockout mice exposed to I/R (76).

It is well-known that elderly patients are more sensitive to ischemic injury. A study demonstrated that METTL3 levels were decreased in both young and elderly mouse ischemic hearts. However, FTO was only decreased in ischemic elderly hearts, and the downregulation of this demethylases may equate with more levels of methyltransferases in aging mice hearts after acute I/R injury, indicating that overall m6A methylation level might be different between young and elderly hearts after acute I/R injury, which might explain the sensitivity to ischemic injury in aging hearts from the epitranscriptomic m6A aspect at least partially (77).

### m6A in Myocardial Infarction

Cheng et al. (78) demonstrated that m6A modification was remarkably decreased in MI mice hearts, and plays a role in acute MI through tricarboxylic acid (TCA) cycle-based metabolic reprogramming in which ALKBH5 acts as an important modulator. ALKBH5 knockout led to lower serum levels of LDH and creatine kinase-MB (CK-MB) as well as improved systolic and diastolic functions in acute MI mice. Gong et al. (79) showed that both knockout and knockdown of METTL3 led to enhanced cardiomyocyte proliferation, decreased infract size, and improved cardiac function in mice after MI. Mechanically, the silence of METTL3 reduced m6A-mediated pri-miR-143 maturation into miR-143-3p, and thus increased the levels of miR-143-3p targets including Yes-associated protein (Yap) and catenin delta-1 (CTNND1), thereby promoting cardiomyocyte proliferation. It seems to be contradictory that both methyltransferase METTL3 and demethylase ALKBH5 exert a similar destructive effect in MI. It may be attributed to different MI models used or different time points of detection. In addition, Shi et al. (80) showed that WTAP level was significantly decreased in MI heart tissues, and WTAP SNP was significantly correlated with the progression of MI based on GWAS analysis. In addition, FTO, YTHDF3, and ZC3H13 were also differentially expressed in MI tissues. Moreover, according to the expression profiles of m6A regulators, they identified three molecular subtypes of MI with different clinical characteristics, providing novel clues to explore molecular mechanisms of distinct subtypes of MI as well as potential biomarkers for the diagnosis of MI.

### m6A in Cardiac Hypertrophy

Gao et al. (81) found that METTL3 increased m6A modification in poly (ADP-ribose) polymerase 10 (PARP10) mRNA and accelerated its degradation. PARP10 is a mono-ADP-ribosyltransferase and was reported promoting cardiomyocyte hypertrophy by inhibiting the activity of GSK3β a well-known suppressor of cardiac hypertrophy through mono-ADP-ribosylation (82, 83), suggesting that METTL3 may play a protective role in cardiac hypertrophy, and METTL3-PARP10-GSK3β axis could be a therapeutic target for cardiac hypertrophy. However, Lu et al. (84) showed that METTL13 plays a harmful role in cardiac hypertrophy, and upregulation of METTL3 aggravated the Ang II-induced cardiac hypertrophy. In addition, METTL3 was also reported regulating the maintenance of cardiac homeostasis. For example, Dorn et al. (85) showed that m6A level was significantly increased in cardiomyocytes exposed to hypertrophic stimulation. Increased expression of METTL3 promoted spontaneous, compensated cardiac hypertrophy in mice without affecting cardiac function when exposed to stress; whereas cardiac-specific METTL3 knockout induced structural and functional changes similar to HF in mice hearts exposed to stress.

Several m6A “readers” might impede cardiac hypertrophy such as YTHDF2 and IGF2BP2. Xu et al. (86) showed that the expression of YTHDF2 was increased in cardiac hypertrophy, and YTHDF2 inhibited cardiac hypertrophy through m6A-dependent mRNA degradation of *MYH7*, a cardiac hypertrophy marker. Qian et al. (87) showed that IGF2BP2 recognized m6A modification in miR-133a target sequence, and then formed a complex with argonaute 2 (AGO2) to facilitate miR-133a’s accumulation on target mRNAs, thereby preventing cardiac hypertrophy.

### m6A in Heart Failure

A recent study showed that m6A level was increased in failing human hearts, and differentially expressed m6A-regulated mRNAs in failing hearts compared to non-failing hearts were most enriched to histone modification. It also identified the conserved m6A-modified transcripts during cardiomyocyte remodeling under stress conditions in humans and rats, and suggested that m6A might have potential influences on post-transcriptional regulation of these conserved transcripts (88). Another study showed that m6A RNA landscape was altered in transverse aortic constriction (TAC)-induced HF mouse models and failing human heart samples, and hyper-methylated transcripts exhibited the increased production of proteins related to cardiac function and metabolic processes. In addition, cardiomyocyte-specific knockout of *Fto* accelerated the progression of HF in TAC mice, manifested by increased dilatation and reduced ejection fraction (89). Mathiyalagan et al. (90) showed that FTO expression was decreased in human and mouse failing hearts. Overexpressing FTO improved Ca^2+^ amplitude, arcomere dynamics, and contractile function in hypoxia-treated cardiomyocytes *in vitro*, and myocardial delivery of FTO attenuated ischemia-induced cardiac dysfunction in failing mice hearts *in vivo*. Shen et al. (70) demonstrated that FTO expression was decreased in heart tissues of TAC- or doxorubicin-induced HF mice. Zhang et al. (91) showed that FTO levels were significantly reduced in both TAC-induced HF mice and HF patients. Overexpression of FTO significantly mitigated cardiac dysfunction by modulating glycolysis and glucose uptake in TAC-induced HF mice. Overall, these studies suggest that FTO might play a protective role in HF.

HF with preserved ejection fraction (HFpEF) accounts for at least 50% of HF; however, its pathogenesis is unclear. Zhang et al. (92) showed that m6A landscape and expression pattern of m6A regulators such as METTL3, METTL4, KIAA1429, FTO, and YTHDF2 were altered in HFpEF, revealing that m6A modification might be responsible for the pathogenesis of HFpEF.

## m6A as Biomarkers for Cardiovascular Diseases

Rapid and timely response to external stimuli confers m6A the potential as the biomarker to dignose or predict CVDs. Pathological features of CVDs might be mirrored to some extent in the peripheral blood epitranscriptome including RNA m^6^A modification. Chang et al. (93) discovered that m6A level was significantly increased at 8 weeks after MI in the heart tissue of SD rats based on the ultra-performance liquid chromatography-tandem mass spectrometry (UPLC-MS/MS) method developed by themselves, and observed a similar change of m6A level in peripheral blood. The result indicates that m6A level in peripheral blood might be related to that in MI heart tissue, providing a potential alternative to heart tissue for the detection of m6A level after MI and MI diagnosis-aided. In addition, Sikorski et al. (94) designed the Ischemic Heart Disease Epitranscriptomics and Biomarkers (IHD-EPITRAN) study to detect the distribution and abundance of RNA m^6^A in peripheral blood through quantitative and qualitative methods, aiming to explore novel biomarkers for IHD from blood.

## m6A-Based Therapy for Cardiovascular Diseases

**FIGURE 2:**
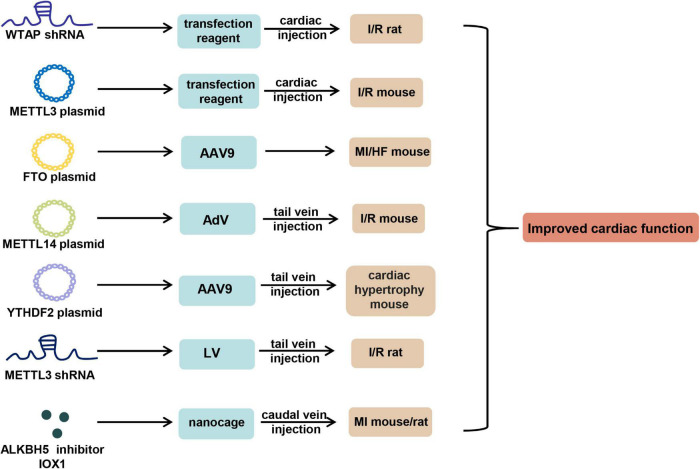
m6A-based therapy for cardiovascular diseases. shRNA of WTAP and METTL3, overexpression plasmid of METTL3, METTL14, FTO, and YTHDF2, and ALKBH5 inhibitor IOX1 can be used to improve cardiac function in mouse or rat models of CVDs by virtue of *in vivo* transfection reagent, AAV9, AdV, LV, and nanocage through the approach of cardiac or tail vein injection. AAV9, adeno-associated virus serotype 9; AdV, adenovirus; LV, lentivirus.

There are three main approaches reported to manipulate m6A for the treatment of CVDs, including transient transfection-based therapy, viral vector-based therapy, and nanometer material-based therapy (Figure 2).

### Transient Transfection-Based Therapy

Wang et al. (72) showed that left ventricular anterior wall injection of WTAP short hairpin (sh) RNA plasmid 24 h before I/R significantly reduced cardiac I/R injury in SD rats. Zhao et al. (73) overexpressed METTL3 in mice myocardial tissues through cardiac delivery of METTL3 overexpression plasmids using *in vivo* transfection reagent, and observed that upregulation of METTL3 significantly improved tissue morphologies in I/R mouse models.

### Viral Vector-Based Therapy

Adeno-associated virus serotype 9 (AAV9) is the most common viral vector for the research of CVDs *in vivo* due to its cardiac tropism. Mathiyalagan et al. (90) constructed FTO plasmids loaded AAV9 system to achieve cardiac gene therapy of *Fto* in mice pre-MI, and observed that cardiac FTO overexpression remarkably improved cardiac function post-MI. In addition, they showed that adenovirus injection of FTO overexpression plasmids post-MI improved cardiac function. Similarly, Zhang et al. (91) demonstrated that overexpressing FTO through AAV9 remarkably alleviated cardiac dysfunction and left ventricular hypertrophy in mice post-TAC. In addition, Pang et al. (74) showed that tail vein injection of METTL14 overexpression plasmids-carrying adenovirus did overexpress METTL14 in mice, and significantly improved cardiac dysfunction during I/R injury. Moreover, Xu et al. (86) showed that administration of AAV9 particles carrying YTHDF2 plasmids through tail vein injection for 3 weeks alleviated cardiac hypertrophy in mice after TAC surgery, indicated by a reduced heart size, decreased hypertrophic cardiomyocytes, and less fibrosis. In addition to these overexpression-mediated therapy, Wang et al. (71) demonstrated that tail vein injection of lentivirus packaged shRNA targeting METTL3 into the I/R rats 24 h before operation significantly alleviated myocardial injury.

### Nanometer Material-Based Therapy

A large C1q molecule was reported bound on dying cells within the infarct area in MI (95), and ALKBH5 could regulate I/R injury through metabolic reprogramming (78). Based on these results, Cheng et al. (78) designed a Mg^2+^ concentration-dependent, ALKBH5 inhibitor IOX1 loaded bioengineered ferritin nanocage modified by C1q ligand Scarf1 on the surface to target dying cells in the area of MI, and the bioengineered ferritin nanocage could phagocytose dying cells by recognizing the transferrin receptor 1 (TfR1) on the surface of dying cells. *In vivo* data showed that daily caudal vein injection of the nanocage for 14 days effectively improved cardiac function in rats or mice post-AMI, indicated by reduced serum levels of LDH and CK-MB, improved LVSP (left ventricular systolic pressure), + dP/dt (maximal rate of increase in left ventricular pressure), and LVEDP (left ventricular end-diastolic pressure), and less fibrillation, providing a potential strategy for AMI treatment in the future.

Altogether, these strategies have their own advantages and limitations. Transient transfection is easily operated and time-saving, but lasts for a short time. AAV9-based gene therapy is long-acting, and has the tissue- or organ-specificity, but implies a risk of genomic integration and activation of immune response. Bioengineered material-mediated therapy seems to be the most promising strategy due to its specificity, stability, security, and high efficiency; however, it is not available to the general scientific communities. To solve the problem, cross-laboratory and cross-disciplinary collaboration is crucial.

## Discussions and Perspectives

Throughout the current studies, it is certain that m6A modification plays a role in CVDs, and the dysregulation of “writers,” “erasers” and “readers” promotes or inhibits the occurrence and progression of CVDs by regulating the metabolism or transfer of targeted mRNAs or non-coding RNAs. m6A level is varied in different types of CVDs. For example, m6A level was reported upregulated in heart failure (88), and downregulated in MI (78). It is because that different CVDs lead to different cell fate transitions and the timely alteration of m6A level enables cells to switch transcriptomes to adapt to the given stimulus or stress. Of note, the upregulation or downregulation of m6A levels may not only be the cause of CVDs, but also be the protective mechanism against the damage from CVDs. In addition, most of the studies reported that the “writer” proteins exerted destructive effects in CVDs, while the “eraser” proteins played protective roles in CVDs; however, several studies still showed the opposite results even for the same “writer” or “eraser” in a given CVD. For example, Wang et al. (71) showed that METTL3 exerted a detrimental role in I/R rats and oxygen-glucose OGD/R-treated cardiomyocytes; however, Zhao et al. (73) demonstrated that METTL3 played a protective role in I/R mice and hypoxia/reoxygenation (H/R) cardiomyocytes. The disagreement may be attributed to different cell or animal models used on one hand. On the other hand, m6A is an extremely dynamic and reversible process, and different time points of detection might lead to inconsistent results. Therefore, future studies should pay more attention to the issue. Moreover, in addition to m6A occurring in mRNAs, it is also intriguing to explore whether the effects exerted by those reported cardiac-specific non-coding RNAs are m6A-mediated. Furthermore, m6A can respond rapidly to external stimuli and thus regulate a class of genes related to a specific type of CVDs. Therefore, combining m6A-seq and RNA-seq/MS may help to excavate the relevant gene classes. Finally, as for the m6A-based therapy for CVDs, developing bioengineered material-based m6A regulation or small molecule m6A agonists and antagonists may have important clinical value in the treatment of CVDs. Of note, the role of m6A effector in CVDs is different in different cell types, tissues, and pathological conditions. Therefore, the clinical application of m6A effector should be designed to be tissue- or cell-specific. Collectively, despite recent advances, current exploitation about m6A in CVDs is only the tip of the iceberg, and more efforts are required to explore the unknown, especially the functions and the underlying mechanisms determining the specificity of m6A effectors in CVDs.

## Author Contributions

CL, LG, WD, QM, NL, and GD contributed to collection of references and manuscript writing. SY and HF contributed to determining the subject and manuscript revisions. All authors contributed to the article and approved the submission.

## Conflict of Interest

The authors declare that the research was conducted in the absence of any commercial or financial relationships that could be construed as a potential conflict of interest.

## Publisher’s Note

All claims expressed in this article are solely those of the authors and do not necessarily represent those of their affiliated organizations, or those of the publisher, the editors and the reviewers. Any product that may be evaluated in this article, or claim that may be made by its manufacturer, is not guaranteed or endorsed by the publisher.
